# Inhibition of Keratinocyte Differentiation by the Synergistic Effect of IL-17A, IL-22, IL-1α, TNFα and Oncostatin M

**DOI:** 10.1371/journal.pone.0101937

**Published:** 2014-07-10

**Authors:** Hanitriniaina Rabeony, Isabelle Petit-Paris, Julien Garnier, Christine Barrault, Nathalie Pedretti, Karline Guilloteau, Jean-François Jegou, Gérard Guillet, Vincent Huguier, Jean-Claude Lecron, François-Xavier Bernard, Franck Morel

**Affiliations:** 1 Laboratoire Inflammation Tissus Epithéliaux et Cytokines, Equipe Accueil 4331, Université de Poitiers, Poitiers, France; 2 Laboratoire Immunologie et Inflammation, Centre Hospitalier Universitaire de Poitiers, Poitiers, France; 3 BIOalternatives, Gençay, France; 4 Service de Dermatologie, Centre Hospitalier Universitaire de Poitiers, Poitiers, France; 5 Service de Chirurgie Plastique, Centre Hospitalier Universitaire de Poitiers, Poitiers, France; CNRS-University of Toulouse, France

## Abstract

Keratinocyte differentiation program leading to an organized epidermis plays a key role in maintaining the first line of defense of the skin. Epidermal integrity is regulated by a tight communication between keratinocytes and leucocytes, particularly under cytokine control. Imbalance of the cytokine network leads to inflammatory diseases such as psoriasis. Our attempt to model skin inflammation showed that the combination of IL-17A, IL-22, IL-1α, OSM and TNFα (Mix M5) synergistically increases chemokine and antimicrobial-peptide expression, recapitulating some features of psoriasis. Other characteristics of psoriasis are acanthosis and down-regulation of keratinocyte differentiation markers. Our aim was to characterize the specific roles of these cytokines on keratinocyte differentiation, and to compare with psoriatic lesion features. All cytokines decrease keratinocyte differentiation markers, but IL-22 and OSM were the most powerful, and the M5 strongly synergized the effects. In addition, IL-22 and OSM induced epidermal hyperplasia *in vitro* and M5 induced epidermal thickening and decreased differentiation marker expression in a mouse model, as observed in human psoriatic skin lesions. This study highlights the precise role of cytokines in the skin inflammatory response. IL-22 and OSM more specifically drive epidermal hyperplasia and differentiation loss while IL-1α, IL-17A and TNFα were more involved in the activation of innate immunity.

## Introduction

The skin is the largest barrier against various physical, chemical or biological stresses, constituting the first line of defense of the body. This physical barrier is constituted of the stratum corneum through protein-enriched cells and lipid-enriched intercellular domains and of the pluristratified nucleated epidermis through tight, gap and adherent junctions, desmosomes and cytoskeletal elements [1]. Cutaneous homeostasis and defenses are controlled by permanent cross-talk amongst dermal fibroblasts, epidermal keratinocytes, and cells of the immune system residing or recruited in the skin. When the skin is stressed, a coordinated inflammatory response is triggered, relayed by specific cytokines. Due to a number of known or lesser known reasons (genetic or environmental factors,…), an inadequate response could generate a cytokine-mediated vicious circle, promoting a chronic inflammation, such as in psoriasis [2].

In this context, keratinocytes are direct targets for numerous cytokines, leading to the regulation of their biological properties contributing to the inflammatory response such as the secretion of cytokines, chemokines and antimicrobial peptides, their differentiation and migration capacities. Nevertheless, it appears that a single cytokine stimulation generates a rather limited effect on keratinocytes, namely, a limited number and/or a limited modulated expression of targeted genes. Since in physiological or physiopathological conditions, tissues are surrounded not by one cytokine but a complex milieu, the study of the biological activities of cytokine combinations is of great interest. A complex cytokine network has been described in psoriasis and highlighted a central role of proinflammatory cytokines such as IL-23, IL-22, IL-17, IL-1 or TNFα produced by infiltrated immune cells [2]–[6]. Cytokine combinations such as IL-17A and IFNγ, IL-17A and TNFα or IL-17A and IL-22 result in a synergistic effect on chemokine and antimicrobial peptide production [7]–[9]. Recently, we showed that the association of IL-1α, IL-17A, IL-22, OSM and TNFα exhibits a very strong synergy on keratinocytes by increasing the expression of inflammatory/innate immunity related molecules such as chemokines and antimicrobial peptides, generating an *in vitro* model of skin inflammation mimicking features of psoriasis [10], [11].

On another hand, changes in epidermal differentiation and lipid composition lead to a disturbed skin barrier which is important for the pathogenesis of skin inflammatory diseases [12]. Genetic linkage of both atopic dermatitis and psoriasis susceptibility to the epidermal differentiation complex on chromosome 1q21, containing more than 30 genes encoding proteins that both build and regulate barrier formation, strongly suggests a role for barrier function or repair in these inflammatory disorders. Barrier disruption stimulates immediate production of cytokines, including TNFα, IFN-γ and IL-1 [13], [14]. This cytokine release controls differentiation and growth of keratinocytes and stimulates local and systemic inflammatory and immune responses. Thus, formation and maintenance of the barrier function is influenced by cytokines. Indeed, psoriasis is usually manifested as raised, erythematous plaques with adherent silvery scales. The scales are a result of a hyperproliferative epidermis and incomplete cornification with retention of nuclei in the stratum corneum. As a result, the epidermis is thickened and, in combination with the dermal inflammatory infiltrate, contributes to the overall thickness of psoriatic lesions. Proinflammatory cytokines are largely involved in this process. IL-22 reduced the expression of numerous keratinocyte differentiation markers (KDM) such as filaggrin (FLG), involucrin (IVL), loricrin (LOR), cytokeratin (CK) 1, CK10, calmodulin-like skin protein (CLSP) and desmocollin 1 (DSC1) [15], [16]. Oncostatin M also affects a similar profile of KDM [17], [18]. Accordingly, both IL-22 and OSM increased thickness of reconstituted human epidermis (RHE) by inducing hyperplasia of the spinous keratinocyte layer. Interestingly, TNFα was also found to inhibit the expression of FLG and LOR by keratinocytes, and treatment of psoriatic patients with TNFα antagonists restored normal expression of FLG and LOR, in correlation with changes in psoriasis area and severity index [19]. In contrast, IL-17A was responsible of a discrete down-regulation of CK10 and LOR expression, but could be implicated in synergic activity with others cytokines [20]. Finally IL-1α has been described to control expression of numerous genes within the epidermal differentiation complex and IL-1α transgenic mice developed a spontaneous skin disease characterized by scaling, hyperkeratosis and parakeratosis, signs of an altered keratinocyte differentiation [21], [22]. Therefore, the effect of more complex cytokine microenvironments on keratinocyte differentiation capacities is raised. Our goal was to study *in vitro* and *in vivo* the activity of proinflammatory cytokine combinations on the keratinocyte differentiation program, including early and late KDM, and to compare our models with KDM expression in psoriatic skin lesions.

## Methods

### Skin samples

The use of human skin samples for research studies was approved by the Ethical Committee of the Poitiers Hospital. The Declaration of Helsinki protocols were followed and patients gave their written informed consent. Biopsies were obtained from the back skin lesions of 5 different patients with moderate to severe plaque psoriasis (mean age = 45 years; PASI>10) that did not receive any therapy for >4 wk. Normal skin biopsies were obtained from surgical samples of healthy breast skin.

### Cell cultures, cytokines and reagents

Normal human epidermal keratinocytes (NHEK) were obtained as previously described, from surgical samples of healthy breast skin [16]. NHEK were cultured to 80% of confluence allowing the expression of a large panel of keratinocyte differentiation markers, and then starved for 24 h in Keratinocyte SFM containing 0.03 mM Ca^2+^ (Invitrogen Life Technologies, Cergy Pontoise, France) before stimulation. Confluent differentiated cells were stimulated with or without recombinant IL-17A, OSM, TNFα, IL-22 and IL-1α alone at maximum effective concentrations (reported previously around 10 ng/ml [16], [17]) or in combination (R&D systems Europe, Lille, France) during 2 h to 72 h for mRNA quantification. RHE were generated on polycarbonate culture inserts, from surgical samples of paediatric foreskins as previously described [23]. RHE were stimulated with or without recombinant IL-17A, OSM, TNFα, IL-22 and IL-1α alone or in combination, with or without a Janus protein Tyrosine Kinases (JAKs) inhibitor 10 µM (Calbiochem, 420099), during 24 h for mRNA quantification or during 72 h for immunohistological analysis.

### In vivo murine skin inflammation

All animal experiments were conducted in accordance with the guidelines and approval of the Institutional Animal Care and Usage Committee at the University of Poitiers. C57Bl/6 mice were purchased from Charles River Laboratories (Chatillon, France). Ear intradermal injections were performed under brief isoflurane (Forene, Abott France, Rungis, France) gas anesthesia. 250 ng of carrier free IL-17A, OSM, TNFα, IL-22 and IL-1α (R&D systems Europe) or PBS were injected in a total volume of 20 µL. After 24 or 48 h, the ears were collected and frozen immediately in liquid nitrogen for H&E staining, immunohistochemistry analysis or mRNA quantification.

### RT-real time PCR analysis

NHEK, RHE and murine skin total RNA were isolated using NucleoSpin RNA II kit (Macherey-Nagel, Hoerdt, France) and reverse-transcribed with SuperScript II Reverse Transcriptase (Invitrogen Life Technologies) according to the manufacturer’s instructions. Quantitative real time PCR was carried out using the LightCycler-FastStart DNA Master^Plus^ SYBR Green I kit on LightCycler 480 (Roche Diagnostics, Meylan, France). The reaction components were 1X DNA Master Mix, and 0.5 µM of HPLC purified sense and anti-sense oligonucleotides purchased from Eurogentec (Eurogentec France, Angers, France), designed using Primer3 software. The stability of the housekeeping gene expression has been assessed by using GeNorm algorithm. The GeNorm software calculates the M value expression stability for the candidate reference genes and considers the gene with the lowest M value to have the most stable expression [24]. The lowest M value for G3PDH demonstrates that the expression is stable under the conditions used for NHEK, RHE and *in vivo* stimulation. Thus samples were normalized to G3PDH housekeeping gene and reported according to the ΔΔC_T_ method as RNA fold increase: 2^ΔΔCT^ = 2^ΔCT sample −ΔCT reference^.

### Histology and immunohistochemistry studies

Six µm cryosection of ears from mice or human skin were fixed in 10% formalin in PBS. Sections of ears were stained with anti-CK10 1:500 (Covance, PBR-159P), anti-LOR 1∶500 (Eurogentec, PRB-145P), anti-FLG 1∶200 (Covance, PRB-417P), anti-CK6 1:250 (ThermoScientific, PA1-29671) and anti-Ki67 1:100 (DakoCytomation) associated with a donkey anti-rat IgG FITC-conjugated secondary antibody or anti-rabbit IgG Rhodamine Red-X conjugated antibody (Jackson Immunoresearch). Cell nuclei were detected with TOPRO 3 1:800 (Invitrogen). Confocal microscopy was carried out on a Olympus FV1000 confocal.

Human skin sections were stained with anti-CK10 1:100 (SantaCruz, SC-23877), anti-LOR 1∶50 (Eurogentec, PRB-145P), anti-FLG 1∶100 (SantaCruz, SC-66192), anti-IVL 1∶20 (Biomedical Technologies, BT-601), anti-S100A7 1:50 (Clinisciences, IMG-409A), and then detected using a biotin-conjugated secondary antibody (Vector, RTU vectastain universal quick kit, PK-7800) and the chromatic substrat AEC (Dako, Substrat hyper-sensible AEC+).

RHE were washed and fixed with formaldehyde solution. Fixed tissues were dehydrated with increasing ethanol concentrations, embedded in paraffin and sections were carried out using a microtome (4 µm thickness). The sections were deparaffinised, washed and incubated with hydrogen peroxide. The sections were incubated with anti-CK10 (SantaCruz, SC-23877), anti-LOR (Eurogentec, PRB-145P), anti-FLG (SantaCruz, SC-66192), anti-IVL (Biomedical Technologies, BT-601), anti-S100A7 (Clinisciences, IMG-409A) and then detected using a biotin-conjugated secondary antibody (Vector, RTU vectastain universal quick kit, PK-7800). After peroxidase-conjugated streptavidine (Vector, RTU vectastain universal quick kit, PK-7800) and peroxidase substrate addition (Dako, Substrat hyper-sensible AEC+), nuclei were counter-stained using a solution of hematoxylin. Sections were observed using a NIKON E400 microscope. The images were captured using a NIKON DS-Ri1 and processed with NIS-Elements 3.10 software.

### Statistics

One-way ANOVA with a Dunnett post-test or Mann-Whitney test were used for the statistical evaluation. The p values were as follows: *p<0.05, **p<0.01, ***p<0.001, and all data are represented as mean and SEM.

## Results

### Synergistic activity of proinflammatory cytokines on inhibition of KDM expression by normal human epidermal keratinocyte

**Figure 1:**
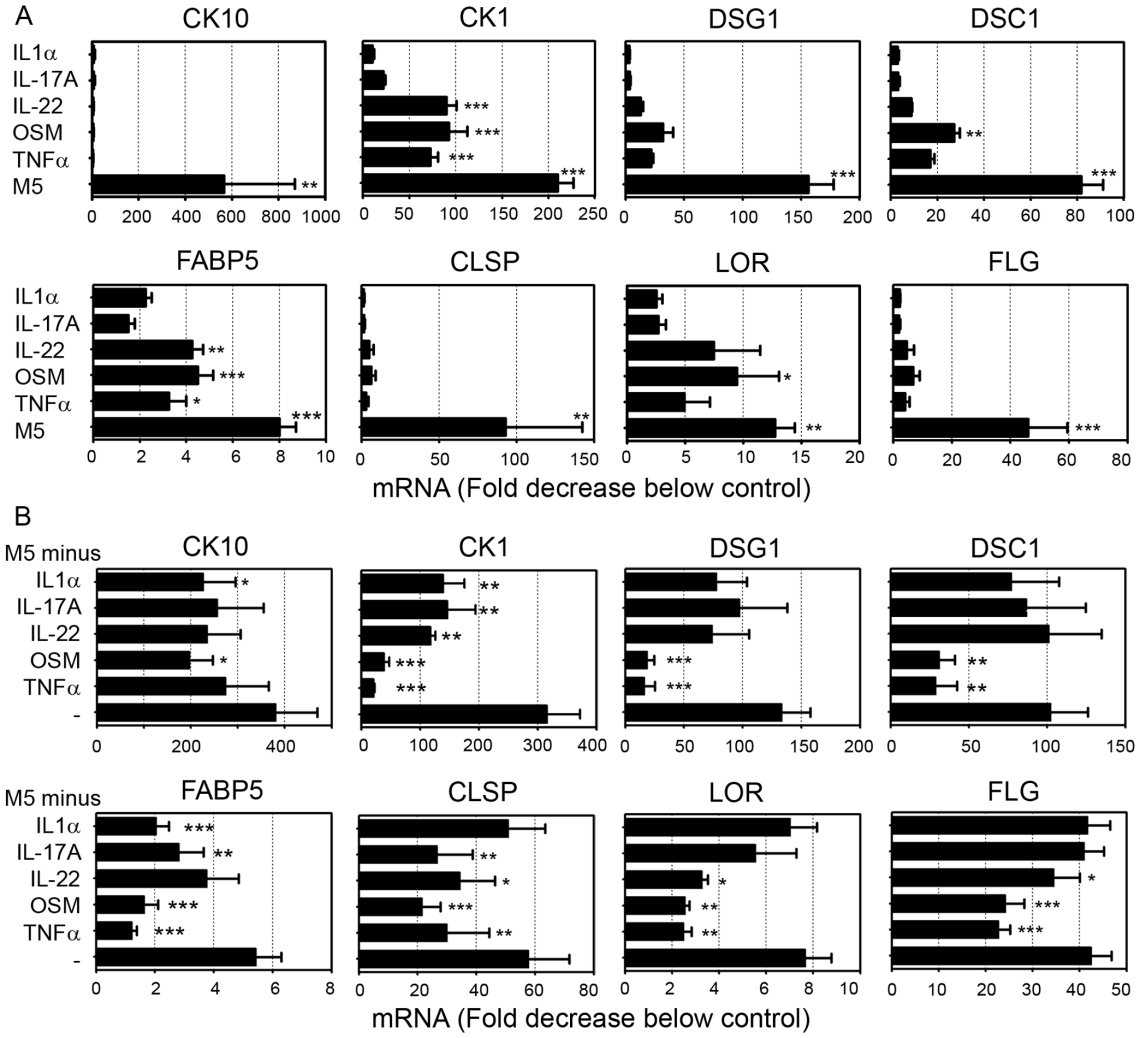
Synergistic activity of proinflammatory cytokines on inhibition of KDM expression by NHEK. NHEK were cultured in the presence or absence of 10/ml of IL-1α, IL-17A, IL-22, OSM and TNFα alone or in combination for 24 h. Quantitative RT-PCR analysis was carried out on total RNA from 4 independent NHEK cultures. mRNA expression levels for cytokeratin 10 (CK10), cytokeratin 1 (CK1), desmoglein 1 (DSG1), desmocollin 1 (DSC1), fatty acid binding protein 5 (FABP5), calmodulin-like skin protein (CLSP), loricrin (LOR) and filaggrin (FLG) were normalized using GAPDH housekeeping gene and expressed as the fold decrease under unstimulated cultures. (A) Comparison of the activity of IL-1α, IL-17A, IL-22, OSM and TNFα alone or in combination (M5) on expression of keratinocyte differentiation markers. (B) Comparison of the activity of mix of 4 cytokines versus mix of 5 cytokines (M5) on expression of keratinocyte differentiation markers. All data are represented as mean and SEM of 4 independent experiments. One-way ANOVA with a Dunnett post-test were used for statistical evaluation and p values were as follows: *p<0.05, **p<0.01, ***p<0.001.

**Figure 2:**
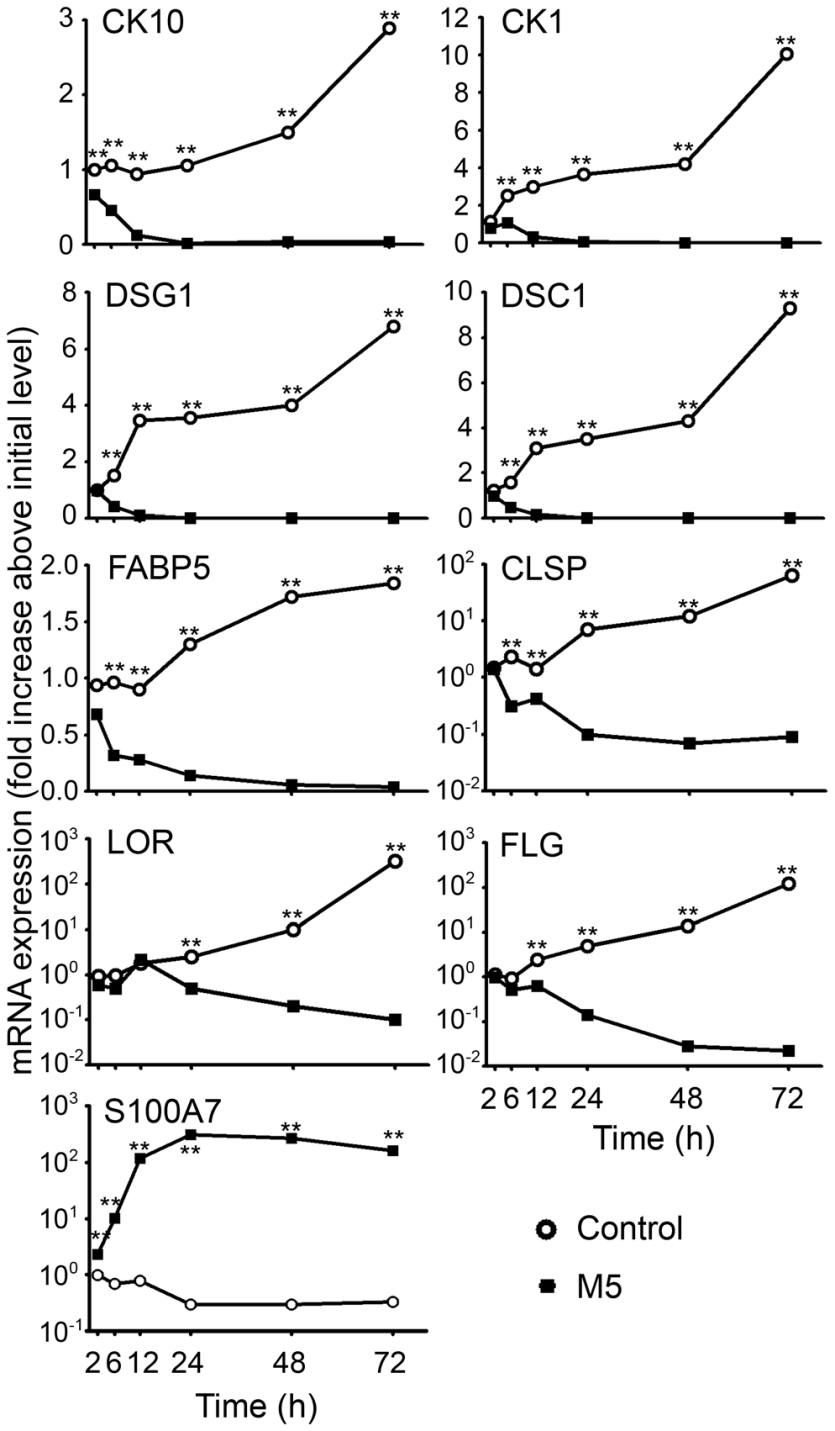
Sustained inhibition of differentiation in NHEK cultured with combination of IL-1α, IL-17A, IL-22, OSM, TNFα. NHEK were cultured in the presence or absence of 10/ml IL-1α, IL-17A, IL-22, OSM and TNFα in combination (M5) for 2 h to 72 h. Quantitative RT-PCR analysis was carried out and mRNA expression levels for cytokeratin 10 (CK10), cytokeratin 1 (CK1), desmoglein 1 (DSG1), desmocollin 1 (DSC1), fatty acid binding protein 5 (FABP5), calmodulin-like skin protein (CLSP), loricrin (LOR), filaggrin (FLG) and S100A7 were normalized using GAPDH housekeeping gene and expressed as the fold increase above initial unstimulated control. Results are from one experiment representative of two. A Mann-Whitney test was used for statistical evaluation and p values were as follows: *p<0.05, **p<0.01.

The activities of IL-1α, IL-17A, IL-22, OSM and TNFα have been studied on KDM expression based on previous reports showing their inflammatory activities on keratinocyte [10], [16], [17]. We previously showed that these cytokines synergistically increased innate immunity, demonstrated by chemokine and antimicrobial peptide production. Since skin inflammation is associated with epidermal hyperplasia, we further asked for such a synergy in keratinocyte differentiation inhibition associated with acanthosis. All five cytokines separately decrease CK10 expression by NHEK between 3 to 8 fold but their combination (M5) results in a strong synergy with a 500 fold decrease of CK10 mRNA expression (Figure 1A). These effects are more varied for other KDM. IL-22, OSM and TNFα downregulate mRNA expression of CK1, desmoglein 1 (DSG1), DSC1, FLG, CLSP, LOR and fatty acid binding protein 5 (FABP5) whereas IL-1α and IL-17A only show minor activities. In addition, a strong synergy of the M5 cytokine combination was observed for DSG1, CLSP and FLG mRNA inhibition, whereas only an additive effect of the cytokines was seen for LOR, DSC1, CK1 and FABP5 (Figure 1A). By removing a single cytokine from the M5 combination, we further identified the major contributors for keratinocyte differentiation inhibition. The absence of OSM or TNFα in the M5 partially restores the control mRNA expression of FLG, CLSP, DSG1, LOR, DSC1, CK1 and FABP5 (Figure 1B), demonstrating that OSM and TNFα were the most potent cytokines for keratinocyte differentiation inhibition. Removal of IL-22, IL-17A or IL-1α partially restores the control mRNA expression of respectively 4, 3 and 3 KDM (Figure 1B). Finally, a kinetic study shows the confluence-induced expression of KDM during culture of unstimulated NHEK, whereas KDM expression under M5 treatment strongly and steadily decreased along culture time when compared to initial expression level (Figure 2). In conclusion, the M5 combination displays a strong and sustained inhibition of keratinocyte differentiation. S100A7 expression under M5 stimulation was strongly induced as early as 6 h and sustained during 72 h illustrating the strong inflammatory response obtained (Figure 2).

### Activity of proinflammatory cytokines on RHE

**Figure 3:**
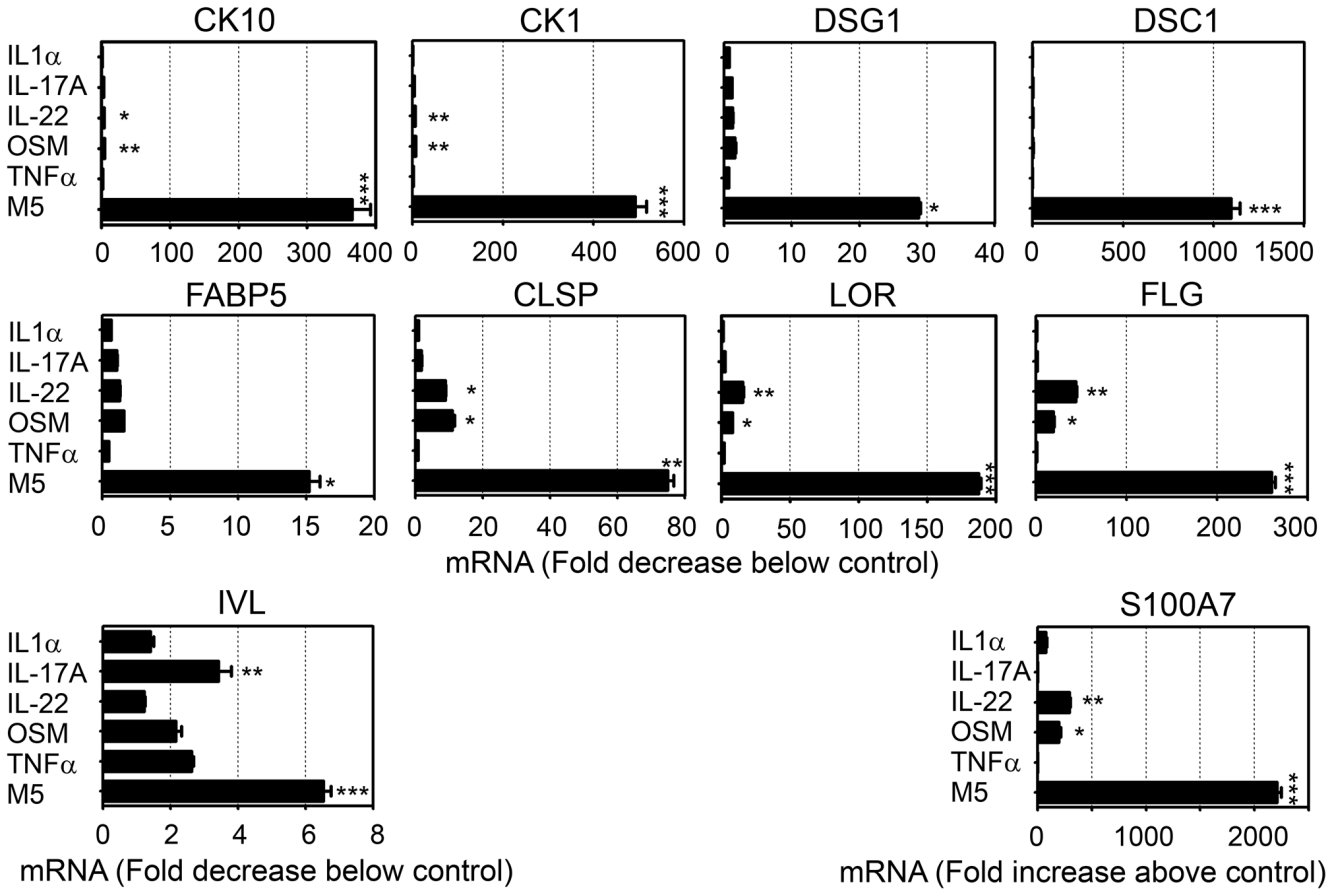
Synergistic activity of proinflammatory cytokines on inhibition of KDM expression by Reconstituted Human Epidermis. RHE have been cultured for 10-water interface using an appropriate differentiation medium and then with or without recombinant IL-1α, IL-17A, IL-22, OSM or TNFα alone or in combination during 24 h for mRNA quantification. Quantitative RT-PCR analysis was carried out and expression levels for KDM were normalized using GAPDH housekeeping gene and expressed as the fold to unstimulated control cultures. Data are mean and SEM of one experiment representative of two. One-way ANOVA with a Dunnett post-test were used for statistical evaluation and p values were as follows: *p<0.05, **p<0.01, ***p<0.001.

In order to confirm the activity of proinflammatory cytokines in a more complete tridimensional model of epidermal differentiation, RHE have been cultured for 10 days at the air-water interface using an appropriate differentiation medium and then stimulated for 24 h or 72 h with the cytokine alone or in combination, before mRNA and protein quantification. Quantitative RT-PCR analysis confirmed that IL-22 or OSM are the most active cytokines to decrease expression of both early and late KDM such as CK10, CK1, LOR and FLG. We also observed a strong synergistic inhibitory effect of the 5 cytokines on all KDM mRNA expression (Figure 3). IVL mRNA expression was discretely inhibited by IL-17A and by the M5, while S100A7 mRNA expression was strongly induced by OSM, IL-22 and synergistically by M5 (Figure 3), as previously described [10].

**Figure 4:**
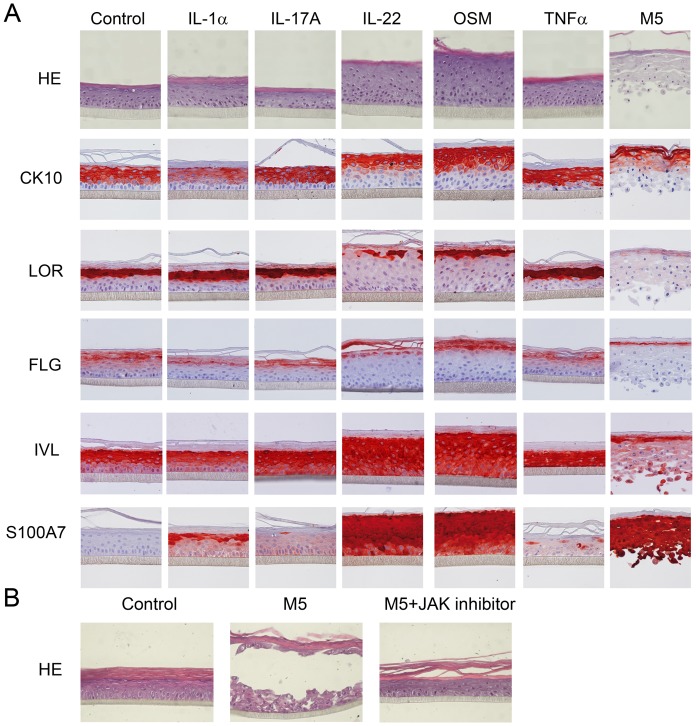
Activities of proinflammatory cytokines on the differentiation of Reconstituted Human Epidermis. (A) RHE have been cultured for 10 days at the air-water interface using an appropriate differentiation medium and then with or without recombinant IL-1α, IL-17A, IL-22, OSM or TNFα alone or in combination during 72 h for immunohistological analysis. RHE were fixed, embedded in paraffin and 4 µm vertical sections were stained with Hematoxylin and Eosin (HE) or with anti-CK10, anti-LOR, anti-FLG, anti-IVL or anti-S100A7 mAbs. Results are from one experiment representative of two. (B) RHE have been cultured for 10 days at the air-water interface using an appropriate differentiation medium and then with or without recombinant IL-1α, IL-17A, IL-22, OSM and TNFα (3 ng/ml), with or without JAKs inhibitor (10 µM) during 72 h. RHE were fixed, embedded in paraffin and 4 µm vertical sections were stained with Hematoxylin and Eosin. Results are from one experiment representative of three.

If IL-1α, IL-17A or TNFα does not modify RHE histology, OSM or IL-22 induces a significant keratinocyte hyperplasia (p<0.001 and p<0.01 respectively) and a loss of keratohyalin granules in the granular layer (Figure 4A). Immunohistological analysis confirmed that OSM or IL-22 decreases expression of CK10, LOR and FLG by RHE, whereas IL1α, IL-17A and TNFα did not exhibit any activity (Figure 4A). On another hand, IVL expression was discreetly induced by IL-17A or TNFα, and strongly by OSM or IL-22. Finally S100A7 was strongly induced by OSM or IL-22, in a lesser extent by IL-1α and very slightly by IL-17A or TNFα. IL-22 or OSM-induced hyperplasia could not be explained by an increased keratinocyte proliferation evaluated using Ki67 staining (data not shown), in agreement with other groups and us [16], [25],

To confirm the synergy observed in NHEK, we stimulated RHE during 3 days with M5. The use of the maximum effective concentrations for each cytokine in M5 results in a complete loss of the integrity of RHE. The use of suboptimal concentrations of each cytokines in M5 (3 ng/ml) is less drastic, showing especially a disruption of the granular layers, associated with a strong inhibition of CK10, LOR, FLG and IVL expression, but sustained S100A7 expression (Figure 4A). The effect of the M5 on RHE disorganization was not due to a direct toxicity of the cytokine mixture on keratinocytes since toxicity has neither been observed in the NHEK model (data not shown). Moreover, a JAKs inhibitor protects the integrity of the RHE and blocked the epidermal hyperplasia, demonstrating that the biological activities of the M5, especially mediated by the JAK-STAT signaling cytokines, were specifically responsible for the tissue disruption (Figure 4B).

### *In vivo* keratinocyte differentiation inhibition by M5 proinflammatory cytokines

**Figure 5:**
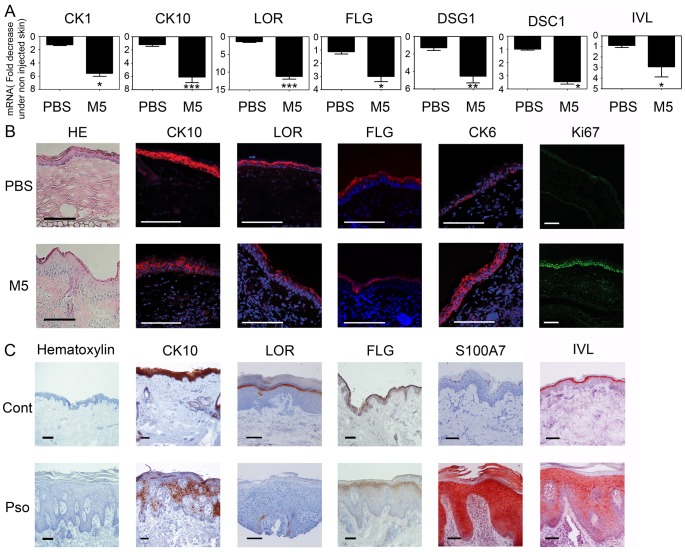
Inhibition of KDM expression *in vivo*. (A) Ears from C57Bl/6 mice were injected intradermally with 250 ng of IL-1α, IL-17A, IL-22, OSM and TNFα (M5) or with Phosphate Buffered Saline (PBS). At 24 h quantitative RT-PCR analysis was carried out on total RNA and expression levels for cytokeratin 1 (CK1), cytokeratin 10 (CK10), loricrin (LOR), filaggrin (FLG), desmoglein 1 (DSG1), desmocollin 1 (DSC1) and involucrin (IVL) were normalized using GAPDH housekeeping gene and expressed as the fold decrease under non injected skin. Data are represented as mean and SEM of 3 independent experiments. *p<0.05, **p<0.01, ***p<0.001. (B) On day 2, the ears were collected for staining with Hematoxylin and Eosin (HE) and immunodetection of cytokeratin 10, loricrin, filaggrin, cytokeratin 6 and Ki-67. Scale bar 100 µm. Results are from one experiment representative of three. (C) Skin biopsies from normal control skin (Cont) or lesional psoriatic skin (Pso) were collected. Skin sections were stained with Hematoxylin and immunodetection of cytokeratin 10, loricrin, filaggrin, involucrin and S100A7 was performed. Scale bar 100 µm. Results are from one experiment representative of three.

To assess *in vivo* the effect of the pro-inflammatory cocktail, the M5 cytokine combination was injected intradermally into the ears of mice. After 24 h, a clear inhibition of CK1, CK10, LOR, FLG, IVL, DSG1 and DSC1 mRNA expression was observed in M5 compared to PBS-injected skin (Figure 5A). Histological analysis performed at 48 h revealed an important epidermal hyperplasia in ears injected with M5 (Figure 5B). Immunohistological analysis confirmed the decreased expression of CK10, LOR and FLG in M5-injected skin (Figure 5B). In parallel, we detected in M5-injected skin a strong expression of CK6 and Ki67 staining revealing an enhanced keratinocyte proliferation under M5 stimulation.

In order to evaluate the pathophysiological relevance of our *in vitro* and *in vivo* models, we analysed the expression of several KDM in normal skin and psoriatic skin lesions. We observed a decreased CK10, LOR and FLG but increased IVL expression in psoriatic skin lesions compared to normal skin (Figure 5C). Finally, S100A7 overexpression in psoriatic lesions was illustrated as a positive control of skin inflammation.

## Discussion

Our results confirmed that, amongst this complex pro-inflammatory cytokine milieu, IL-22, OSM and TNFα play a central role in the down-regulation of FLG, CLSP, DSG1, LOR, DSC1, CK1, CK10 and FABP5 expression. Several cytokines control many key components of the stratum granulosum/corneum and appear to have the capacity to profoundly affect skin differentiation. However cytokine synergy has not been extensively studied in this context. Indeed the strong synergy described for IL-17A, IL-22, OSM, TNFα and IL-1α in the upregulation of chemokines and antimicrobial peptides is also true for the inhibition of keratinocyte differentiation, as evidenced by the strong decrease of CK10, CK1, DSG1, DSC1, FLG, LOR or CLSP expression by RHE. Molecular mechanisms underlying this synergy are probably related to the concomitants signaling pathways activated by these cytokines. Stat3 activating cytokines, OSM and IL-22, are particularly important for the control of keratinocyte differentiation [16], [17], as confirmed by the inhibition of the cytokine induced-hyperplasia by the JAKs inhibitor in the present study. Furthermore, in transgenic mice with keratinocytes expressing a constitutively active Stat3, suprabasal CK1 was decreased and replaced by CK6, suggesting an alteration of keratinocyte differentiation, as observed in human psoriasis [26]. CCAAT/enhancer binding proteins should also be considered as they were implicated in IL-17 signaling, and are coordinately regulated as keratinocytes exit the basal layer and undergo terminal differentiation [27], [28]. In addition, the activation of the c-Jun N-terminal kinases dependent pathway is involved in the TNFα dependent modulation of FLG and LOR expression [19]. Finally, even if NF-κB involvement in epidermal proliferation, differentiation and function has been described, the role of NF-κB activation by IL-1, TNFα or IL-17A in the context of inhibited keratinocyte differentiation should be analyzed in more details. Kinetic studies and the evaluation of RHE differentiation status showed that the M5 cytokine combination provided a strong and sustained inhibition of keratinocyte differentiation, leading to both hyperplasia and partial disruption of the epidermis. This was accompanied by a reduction of 10 to 1000 fold of the expression of all the differentiation markers analyzed. As previously described, RHE acanthosis induced by OSM or IL-22 could not be explained by increased keratinocyte proliferation [16], [17], [25]. We hypothesize that epidermal hyperplasia obtained on RHE was mainly due to the inhibition of differentiation and to the prolonged life of keratinocyte before terminal different in corneocytes. To characterize the destructive effect observed in the RHE at the maximum effective cytokine concentrations, a particular attention will be paid to the expression patterns of desmoglein, desmocollin and other desmosomal glycoprotein components. The role of JAK-STAT signaling cytokines will be particularly studied, since a JAKs inhibitor completely blocked the tissue destruction. On the contrary, IVL expression was downregulated *in vitro* by M5 but largely increased *in vivo* at the protein level in the psoriatic lesions, suggesting a post-transcriptional regulation and/or involvement of other cytokines in the regulation of the keratinocyte differentiation.

Injection of the 5 cytokines in the ears of mice also resulted in acanthosis associated with a decrease of KDM expression and increased keratinocyte proliferation illustrated by Ki-67 staining. Thus the epidermal hyperplasia observed *in vivo* resulted from both an altered differentiation and an increased proliferation, as reported for human psoriatic lesions [2]. This was also observed in other mouse models of psoriasis as the K5-Stat3C mice or imiquimod-treated mice [29]. Interestingly, using the same five-cytokine injection model, we previously described a strong inflammatory response associated with chemokine and anti-microbial peptide expressions [10]. Taken together, we can recapitulate many aspects of psoriatic lesions by using this synergistic cytokine cocktail, with defined specific functions for each of these cytokines. If IL-1α, IL-17A and TNFα were important for the production of antimicrobial-peptides and chemokines, IL-22, OSM appears essential to the differentiation inhibition. Finally, numerous clinical trials showed that targeting one cytokine, such as TNFα or IL-17A, is a successful therapeutic strategy for psoriatic patients [30]. Such cytokine inhibition approach could lead to the break of synergy and explain their spectacular efficacy. Establishment of these *in vitro* and *in vivo* models should clarify the role of cytokines in the establishment of the cutaneous inflammatory response.
